# Family Members' Knowledge and Perspectives on Alzheimer's Disease in People with Down Syndrome

**DOI:** 10.1002/alz70858_104102

**Published:** 2025-12-26

**Authors:** Zihan Yan, Eric Doran, Pamela L. Flodman, Katherine A. Hall, Diana K. Valencia, Ira T. Lott

**Affiliations:** ^1^ University of California, Irvine, Irvine, CA, USA

## Abstract

**Background:**

The increased life expectancy of individuals with Down syndrome (DS) has led to a growing awareness of their higher risk for Alzheimer's disease (AD). However, limited research exists on family members' knowledge of this association (DS‐AD) and their perspectives on acquiring this critical information. This study aimed to assess family members' knowledge of DS‐AD, explore their information‐seeking behaviors, and evaluate their attitudes toward research participation.

**Method:**

The study recruited family members of individuals with DS through collaborations with DS associations and support groups across the United States, direct outreach at DS clinics, and participation in the National DS Congress Annual Convention. Participants were adults residing in the United States who could read English or Spanish. This approach yielded 153 valid survey completions using the validated Alzheimer's Disease Knowledge Scale (ADKS), a newly developed DS and AD Knowledge Scale (DSADKS), the validated Research Attitudes Questionnaire (RAQ), and questions about DS‐AD learning preferences.

**Result:**

Analysis revealed varying levels of AD knowledge, with education and ethnicity emerging as significant factors. Graduate degree holders scored higher than those with high school education (*p* <0.001), and non‐Hispanic participants scored higher than Hispanic participants (*p* <0.0001). DS‐AD awareness timing varied: 36% learned before their loved one reached age 10, 25% learned during ages 11‐30, 18% learned during ages 31‐50, and 9% learned after age 50, while 12% remained unaware. Notably, 31% preferred not to share DS‐AD information with their loved ones with DS. Self‐learning (39%) and DS support organizations (36%) were primary information sources, with healthcare providers accounting for only 9%. Research attitudes remained consistently positive across demographic groups.

**Conclusion:**

These findings highlight the need for targeted educational interventions and culturally sensitive approaches to DS‐AD information delivery. The disassociation between support group participation and knowledge levels suggests support groups might fill a different role than education. The strong preference for self‐learning indicates a need for developing reliable, accessible resources. The research participation rate (55%) has room for improvement through reducing participation barriers and enhancing recruitment approaches. These insights can guide the development of more effective educational strategies and research recruitment approaches for families with DS.

